# A novel danshensu derivative ameliorates experimental colitis by modulating NADPH oxidase 4‐dependent NLRP3 inflammasome activation

**DOI:** 10.1111/jcmm.15890

**Published:** 2020-09-17

**Authors:** Li‐Long Pan, Zhengnan Ren, Yanyan Liu, Yalei Zhao, Hongli Li, Xiaohua Pan, Xin Fang, Wenjie Liang, Yang Wang, Jun Yang, Jia Sun

**Affiliations:** ^1^ Wuxi School of Medicine and School of Food Science and Technology Jiangnan University Wuxi China; ^2^ State Key Laboratory of Food Science and Technology Jiangnan University Wuxi China; ^3^ Department of Medicinal Chemistry School of Pharmacy Fudan University Shanghai China; ^4^ Department of General Surgery and Public Health Research Center Affiliated Hospital of Jiangnan University Jiangnan University Wuxi China

**Keywords:** DSC, inflammatory bowel disease, NADPH oxidase 4, NLRP3 inflammasome, reactive oxygen species

## Abstract

We have previously reported a novel compound [4‐(2‐acetoxy‐3‐((R)‐3‐(benzylthio)‐1‐methoxy‐1‐oxopropan‐2‐ylamino)‐3‐oxopropyl)‐1,2‐phenylene diacetate (DSC)], derived from danshensu, exhibits cytoprotective activities in vitro. Here, we investigated the effects and underlying mechanisms of DSC on dextran sodium sulphate (DSS)‐induced experimental colitis. We found that DSC treatment afforded significant protection against the development of colitis, evidencing by suppressed inflammatory responses and enhanced barrier integrity. Intriguingly, DSC specifically down‐regulated DSS‐induced colonic NADPH oxidase 4 (Nox4) expression, accompanied by a balanced redox status, suppressed nuclear factor‐κB (NF‐κB) and NLRP3 inflammasome activation and up‐regulated nuclear factor (erythroid‐derived 2)‐like 2 and haeme oxygenase‐1 expression. In vitro study also demonstrated DSC also markedly decreased Nox4 expression and activity associated with inhibiting reactive oxygen species generation, NF‐κB activation and NLRP3 inflammasome activation in bone marrow‐derived macrophages. Either lentiviral Nox4 shRNA‐mediated Nox4 knockdown or Nox4‐specific small‐interfering RNA mimicked effects of DSC by suppressing NLPR3 inflammasome activation to alleviate experimental colitis or inflammatory macrophage response. Collectively, our results provide the first evidence that DSC ameliorates experimental colitis partly through modulating Nox4‐mediated NLRP3 inflammasome activation.

## 1 INTRODUCTION

Inflammatory bowel disease (IBD), including ulcerative colitis and Crohn's disease, is a common relapsing inflammatory disorder of the gastrointestinal tract and is clinically characterized by recurrent and long‐lasting episodes of diarrhoea and abdominal pain.^1^, ^2^ Mucosal barrier damage, inflammation homeostasis disruption and inflammatory cell infiltration are associated with decay process of the illness.^3^ Current therapies for IBD depend mainly on anti‐inflammatory drugs, antibiotics or biologics that are either not all effective, of high cost or with adverse effects. Therefore, the development of novel nutraceuticals or therapies for IBD remains imminently needed.^4^

Accumulating evidence suggests that IBD arises from aberrant innate and/or adaptive immune responses due to destroy intestinal homeostasis.^5^ Inflammatory conditions in general have been linked to the overproduction of reactive oxygen species (ROS).^6^ Overproduction of ROS is a hallmark of inflammation and also elevated in clinic and experimental colitis.^7^, ^8^, ^9^ Excessive ROS also further induces inflammatory cytokine (such as interleukin‐6 (IL‐6)) release and enzyme (such as cyclooxygenase‐2 (COX‐2) and inducible nitric oxide synthase (iNOS)) expression in colon. Mounting evidence indicates nicotinamide adenine dinucleotide phosphate (NADPH) oxidase family, a group of membrane enzymes producing ROS, is responsible for pathological progression of colitis.^9^, ^10^, ^11^, ^12^, ^13^ Increased NADPH oxidase expression and activity have also been correlated with the severity of IBD in humans and in animal models.^14^ Thus far, seven NADPH oxidase isoforms (Nox1‐5 and Duox1 and 2) have been identified, which form the basis of distinct NADPH oxidases and have varying requirements for other protein subunits.^15^ Distinct from other NADPH oxidase members, Nox4 is not expressed constitutively in normal tissues but can be rapidly induced in various cell types and tissues, including the colon.^16^, ^17^, ^18^ In addition, Nox4 is constitutively active and does not require cytosolic factors for its activation.^19^ Meanwhile, inhibition or knockdown of Nox4 attenuates inflammatory responses in macrophage and ischaemic myocardium.^16^, ^17^, ^18^, ^20^ Ours as well as other previous studies have demonstrated that enhanced Nox4 appears important in various diseases, including colitis.^13^, ^16^, ^21^, ^22^, ^23^, ^24^ Importantly, it is still unclear to what extent Nox4 is important in mediating the process of colitis.

**Figure 1:**
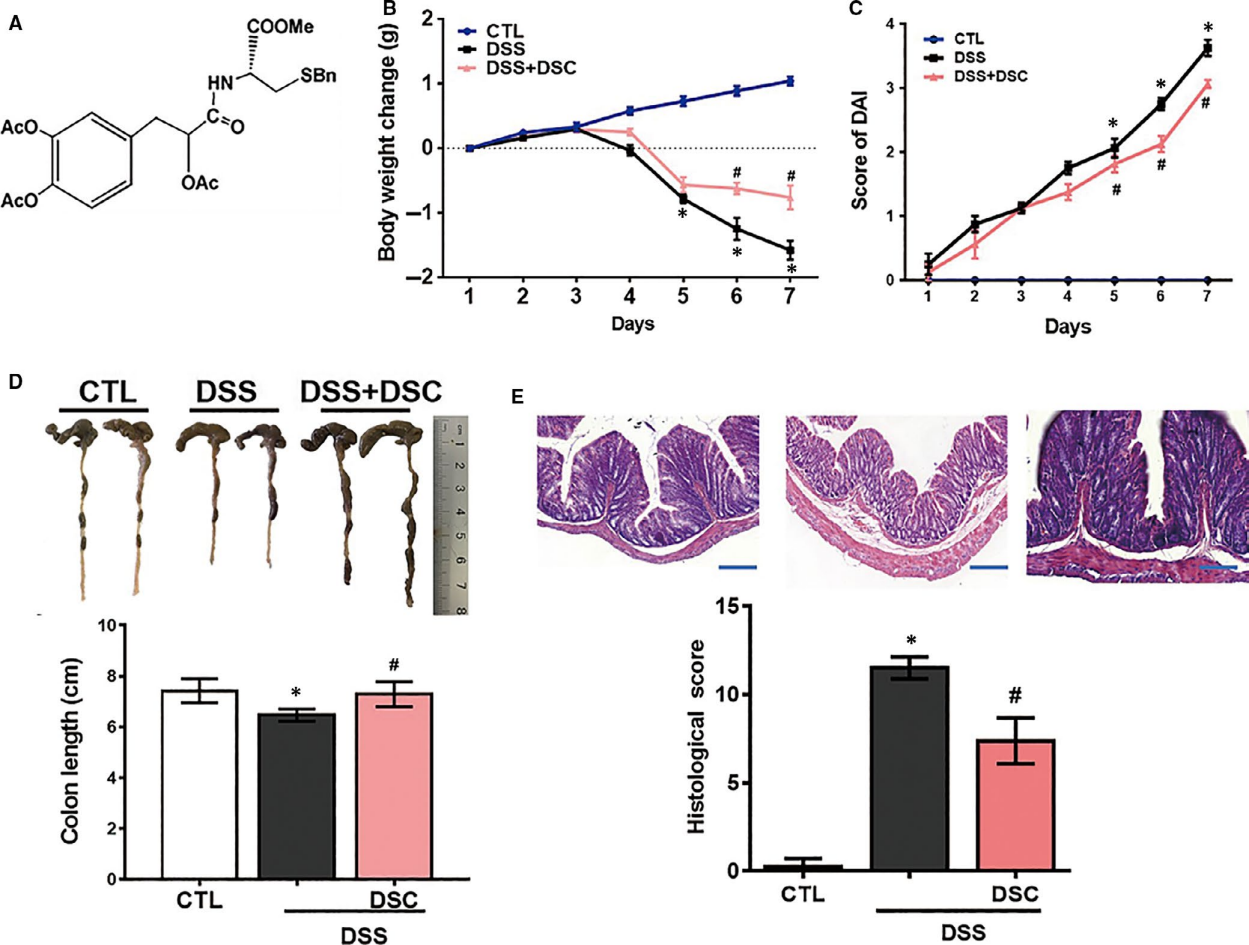
DSC attenuates DSS‐induced experimental colitis. Colitis was induced as described in Materials and Methods and treated with or without DSC (50 mg/kg). A, The chemical structure of DSC. Body weight change (B), DAI score (C), colon length (D), colonic haematoxylin and eosin (H&E) staining and histological score (E) were evaluated in colonic tissue after DSS administration. Data shown are means ± SEM of n = 8 in each group. *P < 0.05 vs Control (CTL), *^#^P* < 0.05 vs DSS‐treated mice

*Salvia miltiorrhiza* Bunge (*S miltiorrhiza*) is a traditional Chinese herbal medicine of the Labiatae family and has been implicated in cardiovascular disorders, blood circulation diseases, neurodegenerative disorders and gastrointestinal inflammation.^25^ *S miltiorrhiza* contains broadly two types of bioactive constituents, hydrophilic phenolic (danshensu, salvianolic acid B, etc) and lipophilic quinines (tanshinone I, tanshinone IIA, dihydrotanshinone I, *etc*).^26^ Danshensu (3‐(3',4'‐dihydroxyphenyl)‐(2R)‐lactic acid) has been demonstrated with various therapeutic effects.^27^ However, its chemical instability of the phenolic hydroxyl groups and relative low abundance in *S miltiorrhiza* limit its application. Our groups have earlier synthesized danshensu derivatives asymmetrically to improve the chemical stability while improving the desired biological functions.^21^ Importantly, we have synthesized and demonstrated its L‐cysteine derivative S‐propargyl‐cysteine with various salubrious biological activities, including anti‐inflammatory, anti‐oxidative and anti‐apoptotic activities.^28^ To further obtain a polyvalent pharmaceutical or nutraceutical candidate, we chemically synthesized a series of novel amide and thioester conjugates of danshensu‐cysteine derivatives by joining two drugs through an appropriate linker or bond in the light of the guidance of medicinal chemical hybridization.^26^ Among them, a novel conjugate [4‐(2‐acetoxy‐3‐((R)‐3‐(benzylthio)‐1‐methoxy‐1‐oxopropan‐2‐ylamino)‐3‐oxopropyl)‐1,2‐phenylene diacetate] (DSC, Figure 1A) has been demonstrated with eminent anti‐inflammatory and anti‐oxidative properties in vitro and in vivo.^26^, ^29^, ^30^ In the present study, we explored the therapeutic potential of DSC in dextran sulphate sodium (DSS)‐induced experimental colitis with special focus on finding novel intracellular molecular target of it.

## 2 MATERIALS AND METHODS

### 2.1 Animals and ethics

Seven‐ to eight‐week‐old male C57BL/6J mice (18‐20 g) were purchased from JOINN Laboratories, Inc (Suzhou, China). They were acclimated for 1 week with tap water and basal diet under the conventional housing conditions of humidity (50 ± 10%), temperature (23 ± 2°C) and light (12/12 hours of light/dark cycle). All animal‐related experimental protocols were approved by the Animal Ethics Committee of Jiangnan University (JN. No 20171215c0300115[90]) in compliance with the recommendations of national and international guidelines for the Care and Use of Laboratory Animals, and were performed in accordance with the guidelines therein.

### 2.2 Induction of colitis and treatment

DSS (MW of 36 000‐50 000) was obtained from MP Biomedicals (Solon, OH, United States). DSC was synthesized by Prof. Yang Wang and its purity was over 96% determined by high‐performance liquid chromatography. Experimental colitis was evoked by DSS (2.5% w/v, *p*.*o*.) in drinking water for 7 days.^31^ The status of the mice was monitored individually by general examination and by measuring body weight loss. In addition, the presence of diarrhoea and haematochezia was noted. Mice were randomly assigned to normal group (drinking regular water), DSS‐treated group (2.5% DSS drinking water), DSC (12.5‐100 mg/kg)‐treated group (DSS‐treated mice also administered DSC, *ip*) (n = 8). DSC was dissolved in 1% dimethyl sulfoxide (DMSO) and administered intraperitoneally at a dosage of 12.5‐100 mg/kg body weight in corn oil once a day. The control group and vehicle group were administered with an equal volume of corn oil with DMSO (1%). For lentiviral transduction, mice received intrarectal instillation of either lentiviral Nox4 shRNA or control shRNA as described previously.^32^ In short, all mice were anaesthetized by sodium pentobarbital (50 mg/kg, *ip*) and given an intrarectal enema of 50% ethanol (vol/vol in distilled H_2_O) before instillation. Pretreatment with ethanol enemas has been shown to increase intestinal transduction with other vectors. Two hours after the enema, 0.1 mL of the concentrated viral suspension with a titre of 1 × 10^9^ TU/mL or vehicle was instilled intrarectally. The mice were inverted for 30 seconds after administration of intrarectal products to prevent leakage. Intrarectal instillation was carried out 3 days before 2.5% DSS stimulation. Mice were randomly assigned to control group, DSS‐treated group, lentiviral control shRNA‐treated group (DSS‐treated mice also administered lentiviral control shRNA), lentiviral Nox4 shRNA‐treated group (DSS‐treated mice also administered lentiviral Nox4 shRNA) (n = 8). At the end of the treatment, mice were killed and the colonic tissues were taken out. The tissues were separated into several parts for extraction of protein and mRNA for subsequent assays. The distal part was fixed in 4% buffered formalin for examination, and other parts were frozen in liquid nitrogen immediately and kept at −80°C until use.

### 2.3 Plasmid construction and lentiviral particle production

Lentivirus generation was performed as our previously described.^21^ The sequences of small hairpin RNA (shRNA) targeting mouse Nox4 were as follows: 5′‐GATCCCTACGCAATAAGAGTTTCTACTCGAGTAGAAACTCTTATTGCGTAGGTTTTTG‐3′.

The scrambled sequence for use as a negative control (sh CTL) is as follows: 5′‐CCTAAGGTTAAGTCGCCCTCGCTCGAGCGAGGGCGACTTAACCTTAGG‐3′. The oligos contain the shRNA sequence flanked by sequences that are compatible with the sticky ends of EcoR I (E. coli RY13 I) (New England Biolabs, Ipswich, MA, USA) and Age I (E. coli strain that carries the cloned and modified AgeI gene from Ruegeria gelatinovora) (New England Biolabs). Forward and reverse oligos were annealed. Then, the pLKO.1 TRC Cloning Vector (Addgene, Watertown, MA, USA) was digested with restriction endonucleases EcoR I and Age I. After that, the Nox4 shRNA insert and linearized plasmid were joined by T4‐DNAligase (New England Biolabs, Ipswich, MA, USA) and ligated into the pLKO.1 TRC Cloning Vector. To obtain the lentivirus, the recombinant plasmid and packaging vectors pD8.2 and pVSVG were cotransfected into 293T cells by Lipofectamine 2000 (Thermo Fisher Scientifics, Shanghai, China). The lentivirus in the culture medium was collected after 48 hours by filtration with 0.45 mm filters and then concentrated via ultracentrifugation for 2 hours at 100 000 g. The viral particles were stored at −80°C.

### 2.4 Scoring of disease activity index

The disease activity index (DAI) was calculated by assigning well‐established and validated scores. Scores were evaluated based on weight loss, stool type and bleeding as described.^33^ All scores were assessed by an independent observer blinded to the treatment.

### 2.5 Macroscopic assessment and histologic analysis

After the mice were killed, colon and spleen tissues were taken out and length of the colon and the wet weight of the spleen were measured. Colonic shortening was determined by measuring the length between the ileocecal junction and the proximal rectum. An independent observer who was blinded to treatment status determined the macroscopic characteristics. Briefly, the macroscopic scores were 0 (no damage), 1 (hyperaemia without ulcers), 2 (hyperaemia and wall thickening without ulcers), 3 (one ulceration site without wall thickening), 4 (two or more ulceration sites), 5 (0.5‐cm extension of inflammation or major damage) and 6‐10 (1‐cm extension of inflammation or severe damage.^34^ For histological evaluation, the colon was collected and fixed in 4% buffered formalin for 36 hours at 4℃, imbedded in paraffin and sliced. 5 μm slices were stained with haematoxylin and eosin (H&E) and examined under a digital slice scanner (Zeiss, Germany) for evaluating the histopathologic changes of the colon tissues. Colonic damage was scored as described previously.^35^

### 2.6 Measurement of myeloperoxidase (MPO) activity

MPO activities were determined with an MPO assay kit (Jiancheng Bioengineering Institute, Nanjing, China) following the manufacturer's instructions. Briefly, fresh colonic tissues were homogenized by a Polytron homogenizer (Scientz‐48, Ningbo, Zhejiang, China) in 50 mmol/L phosphate buffer containing 0.5% hexadecyltrimethylammonium bromide. The homogenates were centrifuged, 20 µL of each supernatant was transferred in duplicate to a 96‐well plate, and 280 µL of 0.02% dianisidine prepared in 50 mmol/L phosphate buffer were added. After 20 minutes incubation at room temperature, absorbance was measured by a spectrophotometer at 460 nm. Protein concentrations of the supernatants were determined by bicinchoninic acid assay. MPO activity was calculated and the value is expressed as units/g.

### 2.7 Cell culture and treatment

Bone marrow‐derived macrophages (BMDM) were derived from tibia and femoral bone marrow cells of C57BL/6 mice and cultured in Dulbecco modified Eagle medium (DMEM) complemented with 10% FBS, 1 mmol/L sodium pyruvate, and 2 mmol/L l‐glutamine in the presence of 20% culture supernatants of L929 mouse fibroblasts.

For inducing interleukin‐1β (IL‐1β), BMDM were plated in a 12‐well plate overnight and the medium was changed to opti‐MEM in the following morning and incubated with DSC (50 μmol/L) for 4 hours, then stimulated with ultrapure LPS (100 ng/mL, InvivoGen, Shanghai, China) for 6 hours, and followed by nigericin (5 μmol/L) treatment for 45 minutes. Cell extracts and precipitated supernatants were analysed by Western blot and ELISA, respectively. In some experiments, BMDM were preincubated with DSC (50 μmol/L, 4 hours) or transfected with Nox4 siRNA and then stimulated with LPS (100 ng/mL) for indicated periods: 1 hour for measurement of nuclear factor‐κB (NF‐κB) p65 phosphorylation, 2 hours for measurement of NF‐κB p65 DNA binding activity, 6 hours for assessment of Nox4 expression and activity, mitochondrial ROS generation, intracellular H_2_O_2_ production, nuclear factor‐erythroid 2 related factor 2 (Nrf‐2) nuclear translocation and haeme oxygenase‐1 (HO‐1) expression.

The nuclear extraction of colonic tissues or BMDM was isolated by using NE‐PER Nuclear and Cytoplasmic Extraction Kit (Thermo Fisher Scientifics, Shanghai, China) according to the manufacturer's instructions. For total protein extraction, colonic tissues or BMDM were lysed in RIPA buffer (Thermo Fisher Scientifics) with protease and phosphatase inhibitor cocktails (Sigma‐Aldrich, Shanghai, China).

### 2.8 Western blot analysis

Western blot analysis was carried out as our previously described.^36^ Equal amounts of protein were separated by electrophoresis in SDS‐PAGE and then transferred onto polyvinylidene difluoride membranes (Millipore, USA). The membranes were blocked with 5% non‐fat dried milk and then incubated with respective primary antibodies overnight at 4°C. After being washed with Tris‐buffered saline with Tween, the membranes were incubated with horseradish peroxidase‐conjugated anti‐rabbit or antimouse secondary antibodies (1:5000, Thermo Fisher Scientifics) for 2 hours at room temperature. The reaction was followed by enhanced chemiluminescence reaction (Thermo Fisher Scientifics), and the blots were quantified using Alpha Imager (Alpha Innotech Corp, San Leandro, CA, USA) with β‐actin or histone 3 as internal control. The results are expressed as fold induction by normalizing the data to the control values. The following antibodies were used: polyclonal rabbit anti‐Nrf‐2 (Cat#16396‐1‐AP, 1:1000, Proteintech Group, Rosemont, IL, USA), polyclonal rabbit anti‐HO‐1 (Cat#10701‐1‐AP, 1:1000, Proteintech Group), COX‐2 (Cat#A1253, 1:1000, Abclonal Biotechnology, Wuhan, China), monoclonal rabbit anti‐Nox4 (Cat#A3656, 1:1000, Abclonal), monoclonal rabbit anti‐β‐actin (Cat#AC026, 1:5000, Abclonal), polyclonal rabbit anti‐Nox4 (Cat#NB110‐58849, 1:500, Novus Biologicals Biotechnology, Shanghai, China), monoclonal rabbit anti‐claudin‐1 (Cat#ab180158, 1:1000, Abcam Biotechnology, Shanghai, China), monoclonal rabbit anti‐caspase‐1 (Cat#ab179515, 1:1000, Abcam), monoclonal rabbit anti‐interleukin‐18 (IL‐18, Cat#ab223293, 1:1000, Abcam), monoclonal rabbit anti‐ZO‐2 (Cat#2847, 1:1000, Cell Signaling Biotechology, Danvers, USA), monoclonal rabbit anti‐NF‐κB p65 (Cat#8242, 1:1000, Cell Signaling), monoclonal rabbit anti‐cleaved IL‐1β (Cat#63124, 1:500, Cell Signaling), monoclonal rabbit anti‐phosphor (p)‐NF‐κB p65 (Cat#3303, 1:1000, Cell Signaling), monoclonal rabbit anti‐nucleotide‐binding domain leucine‐rich‐repeat‐containing family, pyrin domain‐containing 3 (NLRP3, Cat#15101, 1:1000, Cell Signaling), monoclonal rabbit anti‐histone 3 (Cat#9717, 1:1000, Cell Signaling), monoclonal mouse anti‐caspase‐1 p20 (Cat#sc‐398715, 1:500, Santa Cruz Biotechnology, CA, USA) and monoclonal mouse anti‐iNOS (Cat#sc‐7271, 1:500, Santa Cruz).

### 2.9 Cytokine measurement

The levels of IL‐1β and IL‐6 in serum and the levels of IL‐1β in culture supernatant of BMDM were measured by ELISA kits (R&D Systems, Minneapolis, MN, USA) following the standard procedure of the manufacturer. Absorbance was measured at 450 nm with a microplate reader (M1000, TECAN, Austria GmbH, Grödig, Austria).

### 2.10 Real‐time quantitative PCR (qPCR)

Total RNA was isolated from colonic tissues by using Trizol (TaKaRa Biotechnology, Dalian, China) according to manufacturer's protocol. RNA (1 μg) of each sample was reverse transcribed using the reverse transcription system of Takara (TaKaRa) following the manufacturer's instructions. An equal volume of cDNA was used as a polymerase chain reaction (PCR) template for determining the mRNA expression level using SYBR‐Green Quantitative PCR kit (TaKaRa) by iCycler iQ system (Bio‐Rad, Hercules, CA, USA). The relative mRNA levels were normalized to mRNA levels of β‐actin (housekeeping control), and calculations for fold change of each mRNA were made on comparative cycle threshold method (2^−ΔΔCt^). The primers used in this study are provided in Supporting Information Table S1.

### 2.11 Tissue and mitochondrial ROS production measurement

For analysing tissue ROS generation, the redox‐sensitive, cell‐permeable superoxide indicator dihydroethidium (DHE) was used to detect the cellular production of superoxide in vivo.^37^ DHE exhibits blue‐fluorescence in the cytosol until oxidized, primarily by superoxide and to a much less extent other reactive oxygen or nitrogen species. With oxidation, DHE intercalates within cellular DNA, staining the nucleus a bright fluorescent red. Briefly, the fresh colonic tissues were completely embedded in optimal cutting temperature compound and frozen at −80°C for 1 hour, then immediately sectioned at 5 μm and mounted on the slides. Fresh phosphate‐buffered saline containing DHE (5 μmol/L, Thermo Fisher Scientifics, Shanghai, China) was then applied onto each section and incubated for 30 minutes in a light‐protected humidified chamber at 37°C. After washing with phosphate‐buffered saline, the nuclei were stained with the 4’, 6‐diamidino‐2‐phenylindole (DAPI). The fluorescence intensity was detected and quantitated by the confocal laser‐scanning microscopy coupled ZEN software (LSM880, Carl Zeiss Inc, Germany) and represented as the mean fluorescence intensity.

MitoSOX™ Red reagent is live‐cell permeant and is rapidly and selectively targeted to the mitochondria. Once in the mitochondria, MitoSOX™ Red reagent is oxidized by superoxide and exhibits red fluorescence. For measuring mitochondrial ROS production, BMDM in 24‐well plates were pretreated with DSC for 4 hours. After removing the DSC, the cells were stimulated with LPS (100 ng/mL) for 6 hours and then incubated with MitoSOX (5 μmol/L, Thermo Fisher Scientifics, Shanghai, China) in Hanks Balanced Salt Solution for 30 minutes, followed by 3 washes with Hanks Balanced Salt Solution. The fluorescence intensity was detected and quantitated by the confocal laser‐scanning microscopy coupled ZEN software (LSM880, Carl Zeiss Inc, Germany) using excitation/emission of 510/580 nm and represented as the mean fluorescence intensity.

### 2.12 Glutathione (GSH) and glutathione disulphide (GSSG) assay

The tissue GSH and GSSG levels were measured using GSH and GSSG assay kit (S0053, Beyotime Institute of Biotechnology, Shanghai, China) according to the manufacturer's instructions. Briefly, protein removal reagent mol/L solution (70 μL) was added to homogenates of colonic tissues (10 mg). Tissue homogenates were allowed to stand at 4°C for 10 minutes and then centrifuged for 10 000 g at 4°C for 10 minutes. The samples were centrifuged, and the supernatant was subjected to GSH and GSSG assay. The total glutathione level was measured by the 5,5′‐dithiobis (2‐nitrobenzoic acid)‐GSSG recycling assay. The absorbance was measured at 412 nm by a microplate reader (M1000, TECAN, Austria GmbH, Grödig, Austria). The amount of total glutathione in samples was calculated according to the standard curve. After samples and standards were treated with 2‐vinylpyridine to block GSH, the GSSG level was quantified similarly to the total glutathione. The amount of GSH was obtained by subtracting the amount of GSSG from the total glutathione. Protein concentration of the supernatants was determined by bicinchoninic acid assay. Values were normalized to protein concentration and expressed as GSH/GSSG ratio.

### 2.13 Intracellular and tissue H_2_O_2_ measurement

Intracellular and colonic tissue H_2_O_2_ levels were detected using a Hydrogen Peroxide Assay Kit (S0038, Beyotime Institute of Biotechnology, Shanghai, China) according to the manufacturer's instructions. H_2_O_2_ could oxidize Fe^2+^ to Fe^3+^, and then Fe^3+^ react with xylenol orange ina colorimetric reaction that could be further detected by a spectrometer. Briefly, for intracellular H_2_O_2_ measurement, BMDM were collected. Lysate buffer was added to the cells (10^6^ cells/200 μL) and homogenized. The homogenates were centrifuged at 12 000 g at 4°C for 5 minutes and the supernatants were collected for subsequent measurement. For colonic tissue H_2_O_2_ measurement, colonic tissues were homogenized (10 mg/200 μL) and centrifuged at 12 000 g at 4°C for 5 minutes to collect the supernatants for measurement. Finally, 50 μL of the supernatant or standard solution and 100 μL of test solution were added into the 96‐well plate and incubated for 30 minutes at room temperature, and then the absorbance at 560 nm was immediately measured using a microplate reader (M1000, TECAN, Austria GmbH, Grödig, Austria). The intracellular or tissue levels of H_2_O_2_ were calculated from standard concentration curve with triplicate experiments, and the value is expressed as µmol/mg tissue.

### 2.14 Cell transfection

To introduce siRNA into BMDM, the cells were plated in 6‐well or 24‐well plates. Individual siRNAs (30 nmol/L), lipofectamine RNAiMAX and Opti‐MEM were mixed and incubated at room temperature for 5 minutes. The siRNA‐lipofectamine RNAiMAX complexes were added to cells for 24 hours, and the medium was replaced by fresh serum DMEM medium after transfection. Nox4 siRNA (sc‐41587), Nrf2 siRNA (sc‐37049) and control siRNA (sc‐37007) were purchased from Santa Cruz Biotechnology (Santa Cruz, CA, USA). Experiments were performed 72 hours after transfection.

### 2.15 NADPH oxidase and NF‐κB activity measurement

NADPH oxidase activity in BMDM was measured using a commercial NADPH oxidase activity assay kit (#GMS50096.1, Genmed Scientifics Inc, Shanghai, China) following the manufacturer's protocol. Oxidized cytochrome c could be reduced by $O_{2}^{‐}$, which is produced by activated NADPH oxidase, and then the changes of reduced cytochrome c can be further detected at 550 nm to evaluate NADPH oxidase activity. In brief, BMDM were starved and treated as indicated and washed twice with ice‐cold phosphate‐buffered saline. Cells were collected by clear solution, and the total protein was extracted by lysis buffer. Protein concentrations in supernatant were determined by bicinchoninic acid assay. The supernatant was incubated with oxidized cytochrome c at 30˚C for 3 minutes. NADPH oxidase substrate NADPH was added to the reaction mixture and incubated at 30°C for 15 minutes. The dynamic tracings of NADPH‐dependent activity were recorded at 550 nm every minute for 15 minutes with a microplate reader (M1000, TECAN, Austria GmbH, Grödig, Austria). NADPH oxidase activity was expressed as % of enzyme activity compared to that of LPS treatment alone.

The nuclear extracts (10 μg) of BMDM were analysed in duplicate for NF‐κB DNA binding activity using TransAMTM NF‐κB p65 assay kit (#40096, Active Motif, Shanghai, China) following the manufacturer's instructions. The OD_450_ was measured by a microplate reader (M1000, TECAN, Austria GmbH, Grödig, Austria).

### 2.16 Statistical analysis

Data are expressed as means ± SEM. Statistical analysis was performed by one‐way analysis of variance (ANOVA) followed by *Tukey's* post hoc test for multiple comparisons and Student's two‐tailed t test was used for comparing two groups using Stat View of GraphPad Prism (version 6; GraphPad Software Inc, San Francisco, CA, USA). The values of *P* < .05 were considered to indicate a statistically significant difference.

## 3 RESULTS

### 3.1 DSC attenuates DSS‐induced experimental colitis

The optimal dose of DSC for treating mice with DSS‐induced colitis was determined by our preliminary study. Mice induced with DSS‐mediated colitis were treated with varying doses of DSC (12.5‐100 mg/kg). The effects were subsequently evaluated by examining the clinical signs of colitis (including the body weight, colon length and mean score). At doses ranging from 12.5 to 100 mg/kg, DSC dose‐dependently alleviated the development of DSS‐colitis (Figure S1). 50 mg/kg as the lowest dose of DSC showing a significant effect was used for subsequent studies. DSS (50 mg/kg) attenuated weight loss (Figure 1B) and reduced the DAI score based on the severity of diarrhoea and rectal bleeding in DSS‐mediated colitis (Figure 1C). In addition, DSC treatment reversed DSS‐induced shortening of colon length (Figure 1D). Macroscopic analysis revealed that DSC treatment improved the colonic histological score in DSS‐treated mice (Figure 1E). Collectively, these results indicate that DSC attenuates DSS‐induced colonic damage in mice.

### 3.2 DSC ameliorates DSS‐mediated colonic barrier dysfunction and inflammatory response

**Figure 2:**
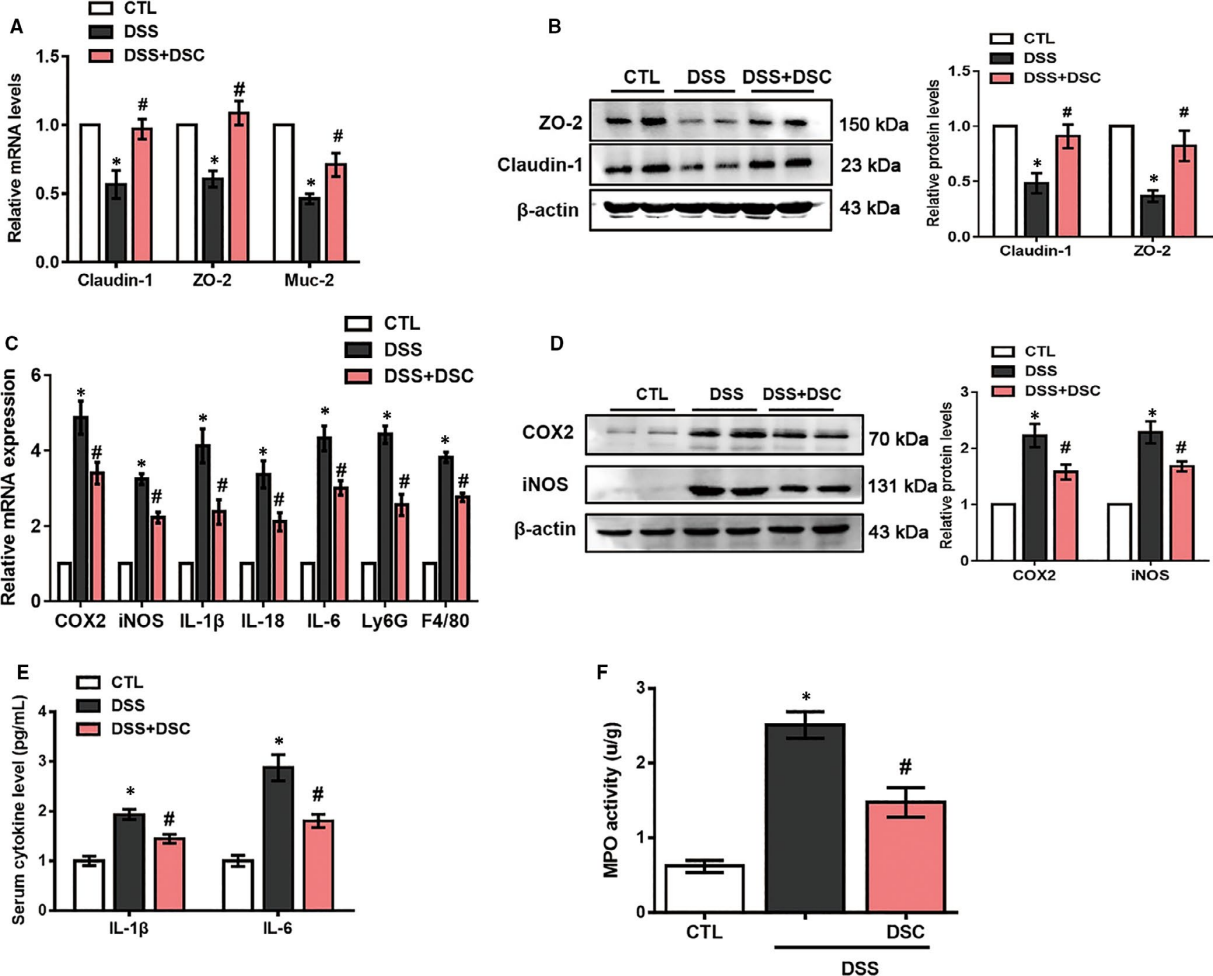
DSC ameliorates DSS‐mediated loss of intestinal barrier integrity and inflammatory response. Colitis was induced as described in Materials and Methods and treated with or without DSC (50 mg/kg). A, The mRNA expression of *claudin‐1, ZO‐2* and *Muc‐2* (*Mucin‐2*) in the colon. B, Representative bands and quantitative analyses of claudin‐1 and ZO‐2 were measured in the colon. C, The mRNA levels of cytokines in colonic tissues. D, Representative bands and quantitative analysis of COX‐2 and iNOS in colonic tissues, β‐actin was used as loading control. E, Serum cytokines. F, colonic MPO activities. Data shown are means ± SEM of n = 8 in each group. *P < 0.05 vs Control (CTL), *^#^P* < 0.05 vs DSS‐treated mice

Subsequently, we evaluated the protective effect of DSC on colonic epithelial mucosal integrity in DSS‐induced colitis. Expression of tight junction proteins (TJP, ZO‐2 and claudin‐1), indicative of intestinal barrier integrity and mucin‐2 (muc‐2) as the intestinal goblet cell marker was examined. DSC administration restored DSS‐induced down‐regulation of ZO‐2 and claudin‐1 at both mRNA and protein levels (Figure 2A,B). Similarly, DSC also induced muc‐2 mRNA expression in experimental colitis (Figure 2A). Inflammatory responses and leucocytes infiltration underpin the development of both experimental and clinical IBD, leading to the mucosal damage.^5^ As shown in Figure 2C, administration of DSC significantly suppressed DSS‐induced mRNA levels of COX‐2, iNOS, IL‐1β, IL‐18, IL‐6, Ly6G and F4/80 in colon, suggesting reduced inflammatory response and innate immune cell (neutrophil and macrophage) infiltration in colon. The effects of DSC on infiltration of macrophage were further confirmed by immunostaining (Figure S2). As shown in Figure 2D,E, DSC treatment also attenuated DSS‐induced elevation of serum IL‐1β and IL‐6 levels and markedly reduced DSS‐mediated colonic COX‐2 and iNOS expression. The effects of DSC on colonic neutrophils were further confirmed by colonic MPO activity in DSS‐colitis mice (Figure 2F). Taken together, these data suggest that DSC ameliorates colitis‐associated disruption of barrier and inflammatory response.

### 3.3 DSC alleviates colonic Nox4‐mediated redox imbalance

**Figure 3:**
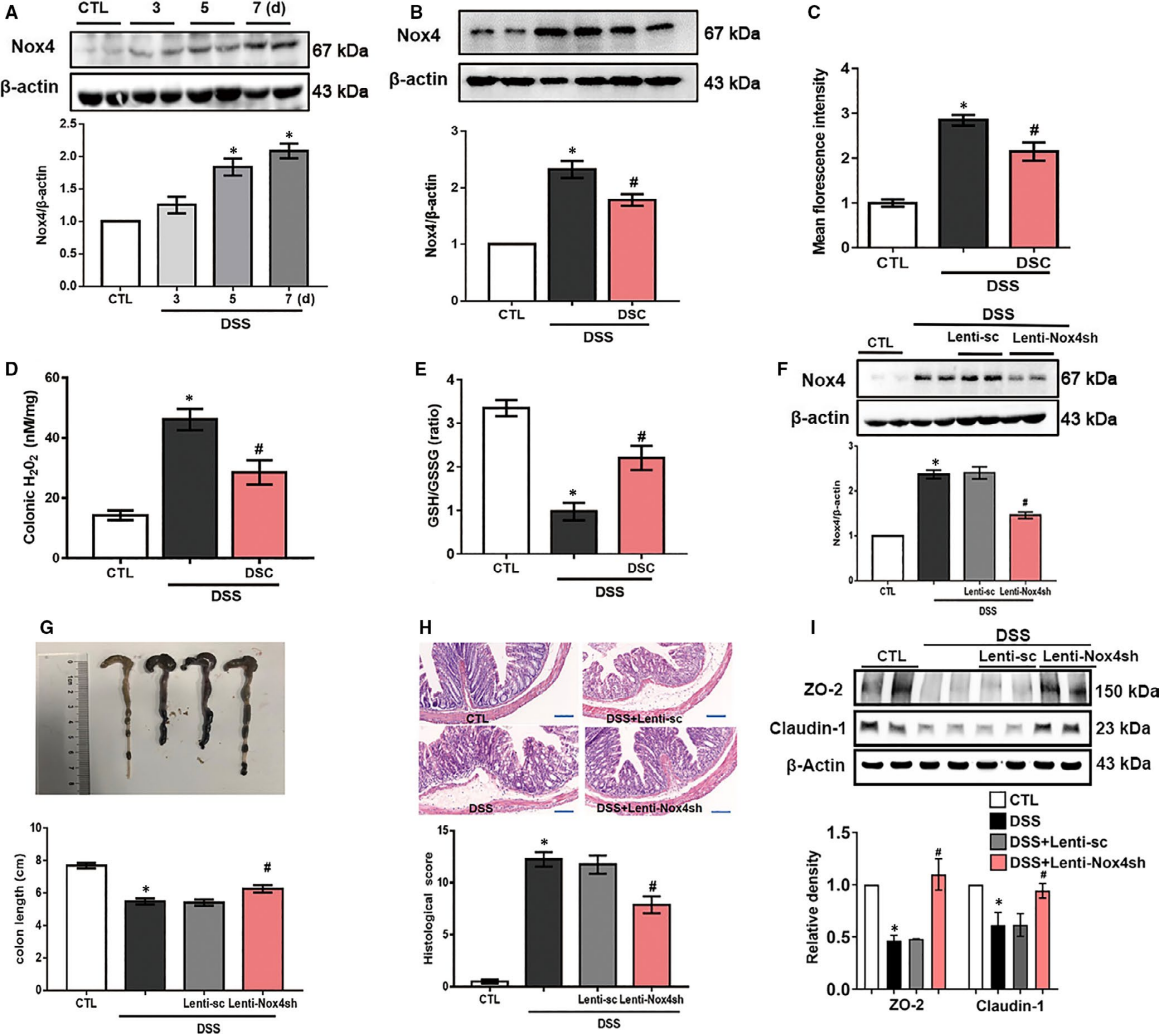
DSC ameliorates Nox4 expression and ROS production. A, Colitis was induced as described in Materials and Methods, and the colonic tissues were collected as indicated periods. Representative bands and quantitative analysis of Nox4 in colonic tissues were shown. B‐F, Colitis was induced as described in Materials and Methods and treated with or without DSC (50 mg/kg). B, Representative bands and quantitative analysis of Nox4 in colonic tissues. C, Representative images and quantitative analysis of ROS production by DHE staining in colonic tissues, scale bar = 100 μm. D, Quantitative analysis of GSH/GSSG ratio. E, Quantitative analysis of tissue H_2_O_2_ production. Colitis was induced as described in Materials and Methods and treated with or lentiviral Nox4 shRNA. F, Representative bands and quantitative analysis of Nox4 in colonic tissues. G, Representative images and quantitative analysis of colon length. H, Representative images of H&E staining and histological score. I, Representative bands and quantitative analysis of ZO‐2 and claudin‐1 expression in colonic tissues. β‐actin was used as loading control. Data shown are means ± SEM of n = 8 in each group. *P < 0.05 vs Control (CTL), *^#^P* < 0.05 vs DSS‐treated mice

NADPH oxidases have been implicated in the pathogenesis of colitis.^9^, ^10^, ^11^ To understand the underlying mechanisms of DSC in suppressing experimental colitis, expression of Nox1, Nox2 and Nox4 in colon was measured. Nox1, Nox2 and Nox4 mRNA expression levels were all elevated in experimental colitis. Intriguingly, DSC specifically reduced DSS‐induced Nox4, but not Nox1 or Nox2 mRNA expression (Figure S3). In addition, Nox4 protein expression was time‐dependently induced in DSS‐treated mice (Figure 3A) and DSC treatment significantly alleviated DSS‐induced up‐regulation of Nox4 at the protein level (Figure 3B). Consistent with modulation of Nox4, DSC treatment also markedly reduced DSS‐induced production of tissue ROS (Figure 3C and Figure S4) and H_2_O_2_ (Figure 3D) and restored the ratio of GSH/GSSG in colon (Figure 3E). To further confirm the role of Nox4 in DSS‐mediated experimental colitis, we delivered lentiviral shRNA to specific Nox4 knockdown in mice. As expected, lentiviral Nox4 shRNA, but not control shRNA, markedly decreased DSS‐induced colonic Nox4 expression (Figure 3F) and mimicked the effects of DSC on alleviating DSS‐induced colitis, as evidenced by attenuated colon length shortening (Figure 3G), improved histological score (Figure 3H), reduced inflammatory mediator (COX‐2 and iNOS) expression, weight loss and MPO activities (Figure S5) and restored TJP (ZO‐2 and claudin‐1) expression (Figure 3I).

**Figure 4:**
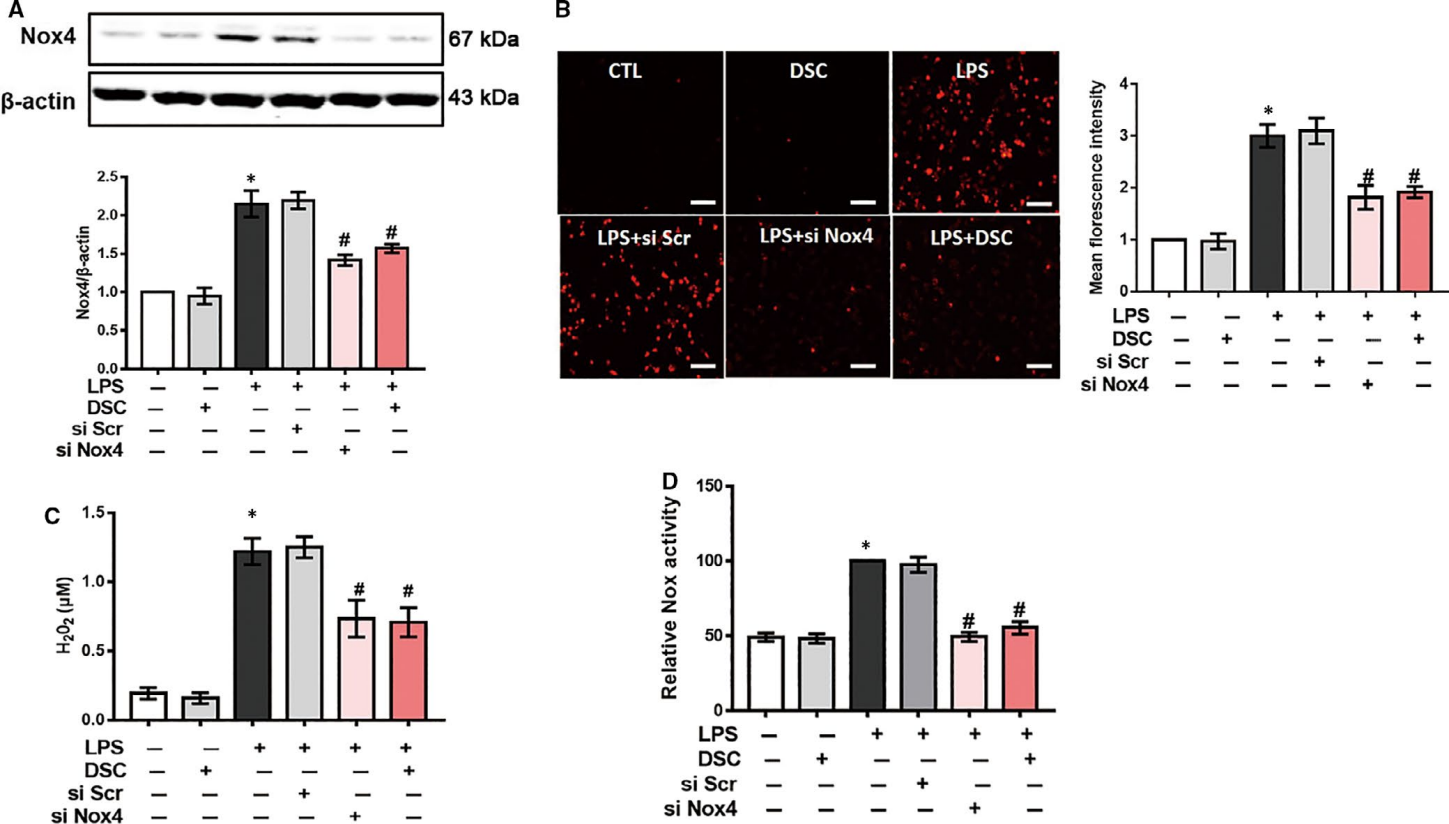
DSC inhibits Nox4‐mediated redox imbalance in BMDM. BMDM was treated with or without DSC (50 μmol/L) or Nox4 siRNA as described in Materials and Methods. A, Representative bands and quantitative analysis of Nox4. B, Representative images and quantitative analysis of mitochondrial ROS production. C, Quantitative analysis of intracellular H_2_O_2_ generation. D, Quantitative analysis of NADPH oxidase activity. Data shown are means ± SEM of n = 8 in each group. *P < 0.05 vs Control cell (CTL), *^#^P* < 0.05 vs LPS‐stimulated BMDM

In line with our previous study,^21^ we also found that Nox4 expression was time‐dependently induced by LPS in BMDM (Figure S6). Transfection with specific Nox4 siRNA, but not scramble siRNA, dramatically reduced LPS‐induced Nox4 expression in BMDM, mimicking DSC (50 μmol/L) pretreatment (Figure 4A). Meanwhile, LPS‐mediated the increase of mitochondrial ROS production, intracellular H_2_O_2_ levels and NADPH oxidase activity in BMDM were markedly attenuated by DSC treatment or Nox4 siRNA transfection (Figure 4B‐D). Taken together, the present results demonstrate that DSC attenuates DSS‐mediated colitis via a Nox4‐dependent mechanism.

### 3.4 DSC inhibits the Nox4‐mediated activation of NLRP3 inflammasome

**Figure 5:**
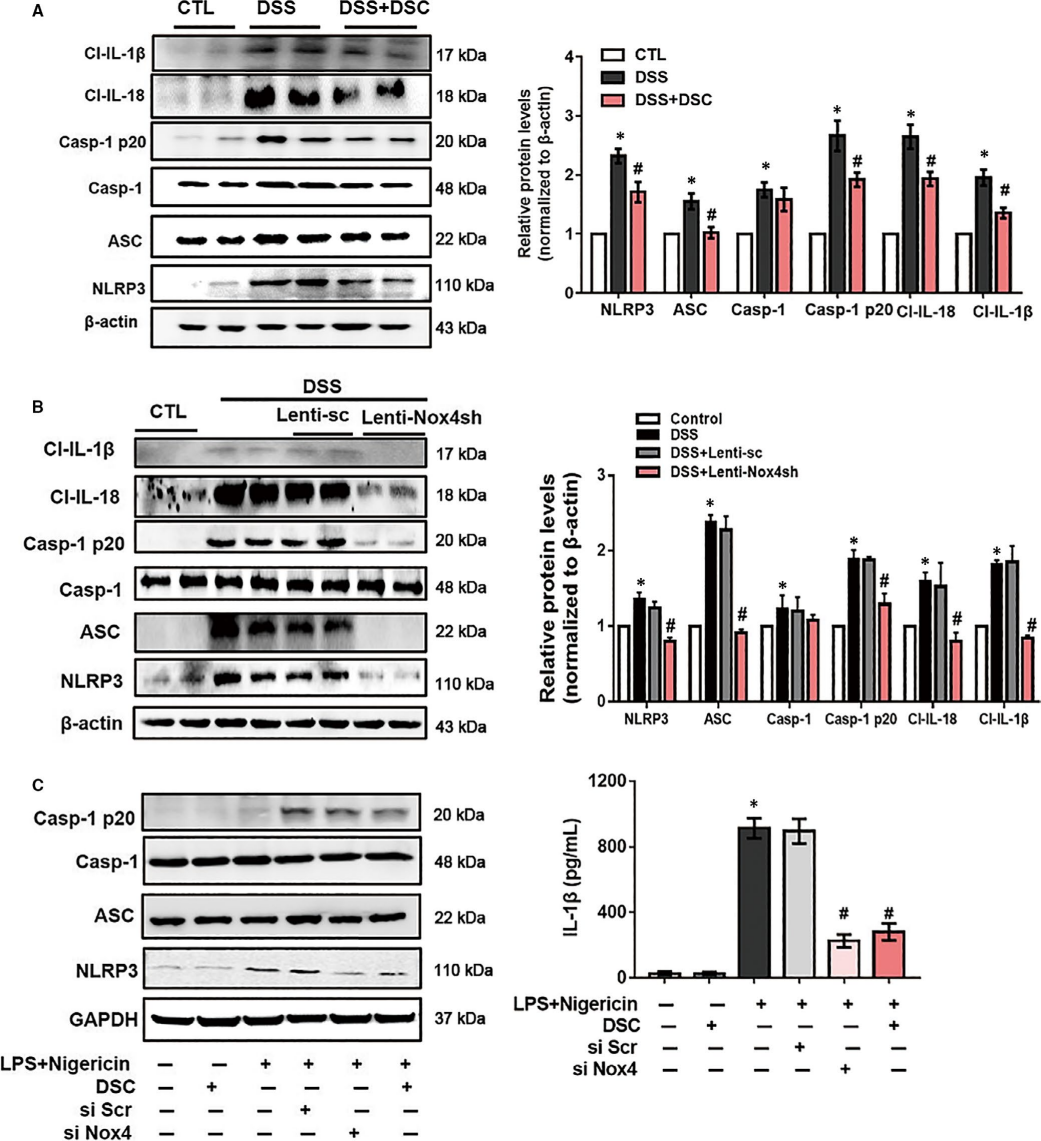
DSC inhibits the activation of NLRP3 inflammasome in vivo and in vitro. A, Colitis was induced as described in Materials and Methods and treated with or without DSC (50 mg/kg). Representative bands and quantitative analyses of NLRP3, caspase‐1 p20, cleaved IL‐1β and cleaved IL‐18 in colonic tissues. The β‐actin was used as loading control (n* = *8 in each group). B, Colitis was induced as described in Materials and Methods and treated with or without lentiviral Nox4 shRNA. Representative bands and quantitative analyses of NLRP3, caspase‐1 p20, cleaved IL‐1β and cleaved IL‐18 in colonic tissues. The β‐actin was used as loading control (n* = *8 in each group). C, NLRP3 inflammasome was induced in BMDM as described in Materials and Methods and treated with DSC (50 μmol/L). Representative bands of NLRP3, ASC and caspase‐1 in BMDM and caspase‐1 p20 in supernatant (SN) by Western blot and quantitative analyses of IL‐1β in supernatant by ELISA. The β‐actin was used as loading control. Data shown are means ± SEM of n = 8 in each group. *P < 0.05 vs Control cell or mice (CTL), *^#^P* < 0.05 vs DSS‐treated mice or LPS + nigericin‐stimulated cells

It is reported that activation of NLRP3 inflammasome plays a critical role in DSS‐induced colitis.^38^ Next, we investigated the effect of DSC on NLRP3 inflammasome in vivo and in vitro. NLRP3 inflammasome was activated after DSS administration, evidencing by up‐regulated NLRP3, ASC caspase‐1 p20, cleaved IL‐1β and cleaved IL‐18 expression in the colon, which was markedly attenuated by DSC treatment (Figure 5A). Up‐regulation of Nox4 promotes NLRP3 inflammasome activation in vitro and in vivo.^20^ As expected, lentiviral Nox4 shRNA, but not control shRNA, mimicked the effects of DSC on alleviating DSS‐mediated NLRP3 inflammasome activation (Figure 5B). To further determine whether Nox4 was involved in the inhibitory effects of DSC on NLRP3 inflammasome activation, we induced activation of NLRP3 inflammasome with nigericin in BMDM primed with LPS. As shown in Figure 5C, DSC (50 μmol/L) alone had no effect on NLRP3 inflammasome activation. Intriguingly, DSC treatment, mimicking Nox4 knockdown, dramatically reduced NLRP3 inflammasome activation and IL‐1β secretion in BMDM. Taken together, these results suggest that DSC suppresses NLRP3 inflammasome activation by modulating Nox4.

### 3.5 DSC inhibits NF‐κB activation and induces Nrf2‐mediated signalling pathways

**Figure 6:**
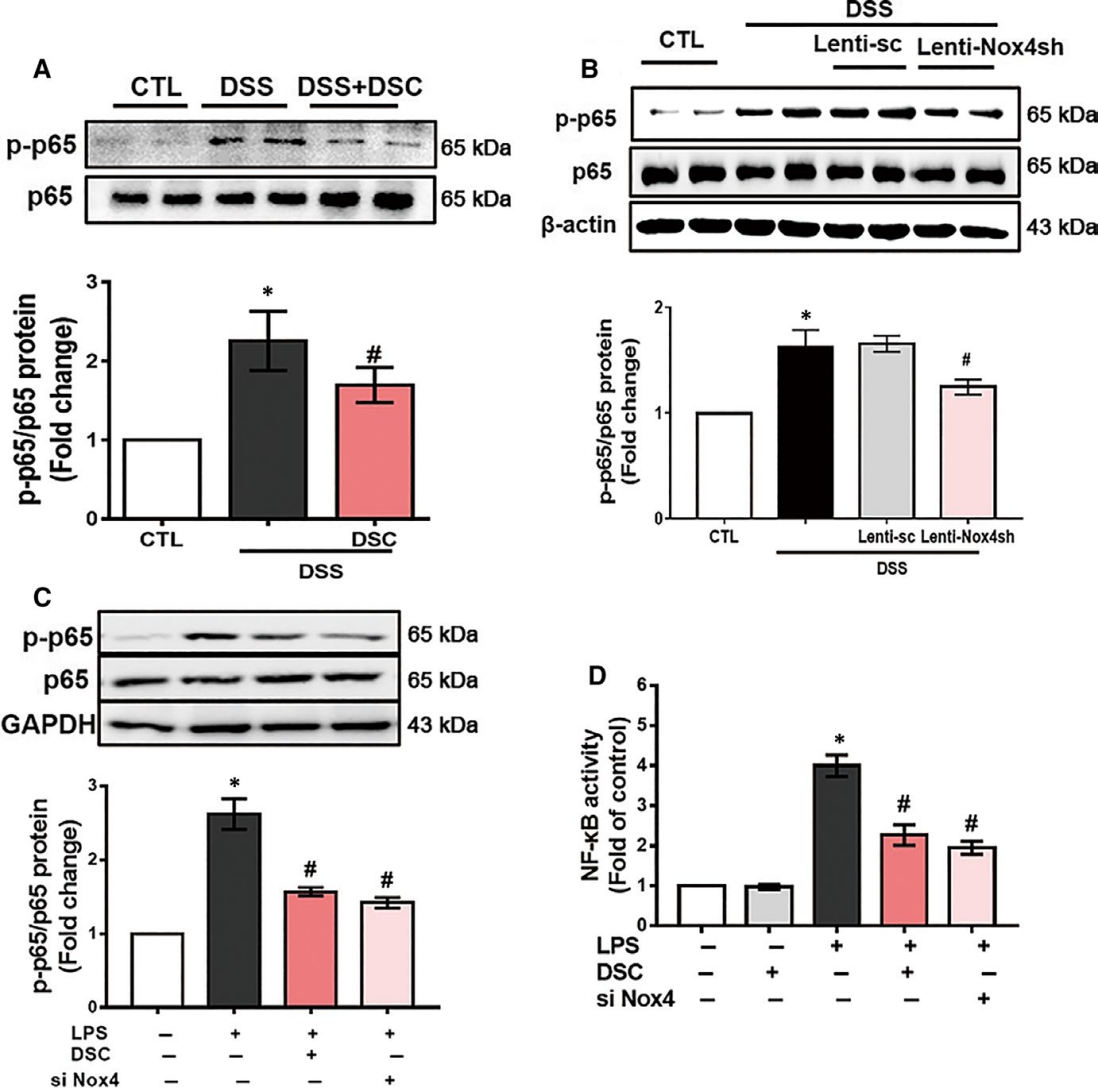
DSC inhibits NF‐κB activation. Colitis was induced as described in Materials and Methods and treated with or without DSC (50 mg/kg). A, Representative bands and quantitative analysis of p‐NF‐κB p65 in colonic tissues, NF‐κB p65 was used as loading control. B, Colitis was induced as described in Materials and Methods and treated with or without lentiviral Nox4 shRNA. Representative bands and quantitative analysis of p‐NF‐κB p65 in colonic tissues, NF‐κB p65 was used as loading control. BMDM was stimulated with or without DSC (50 μmol/L) as described in Materials and Methods. C, Representative bands and quantitative analysis of p‐NF‐κB p65 in BMDM and NF‐κB p65 was used as loading control. D, Quantitative analysis of NF‐κB p65 DNA binding activity in BMDM. Data shown are means ± SEM of n = 8 in each group. *P < 0.05 vs Control cell or mice (CTL), *^#^P* < 0.05 vs DSS‐treated mice or LPS‐stimulated cells

NF‐κB is a transcription factor regulating expression of inflammatory mediators (including NLRP3, COX‐2 and iNOS) and various pro‐inflammatory cytokines, and the effect of DSC on the activation of NF‐κB was evaluated. Phosphorylation of p65 at serine 536 appeared to play a critical role in NF‐κB DNA binding activity.^39^ As shown in Figure 6A, DSC treatment dramatically reduced DSS‐mediated the phosphorylation of NF‐κB p65 (serine 536) in colonic tissues, which was also mimicked by lentiviral Nox4 shRNA (Figure 6B). Similarly, both DSC and silencing Nox4 remarkably suppressed NF‐κB p65 phosphorylation (serine 536) and NF‐κB p65 DNA binding activity in LPS‐stimulated BMDM (Figure 6C,D).

**Figure 7:**
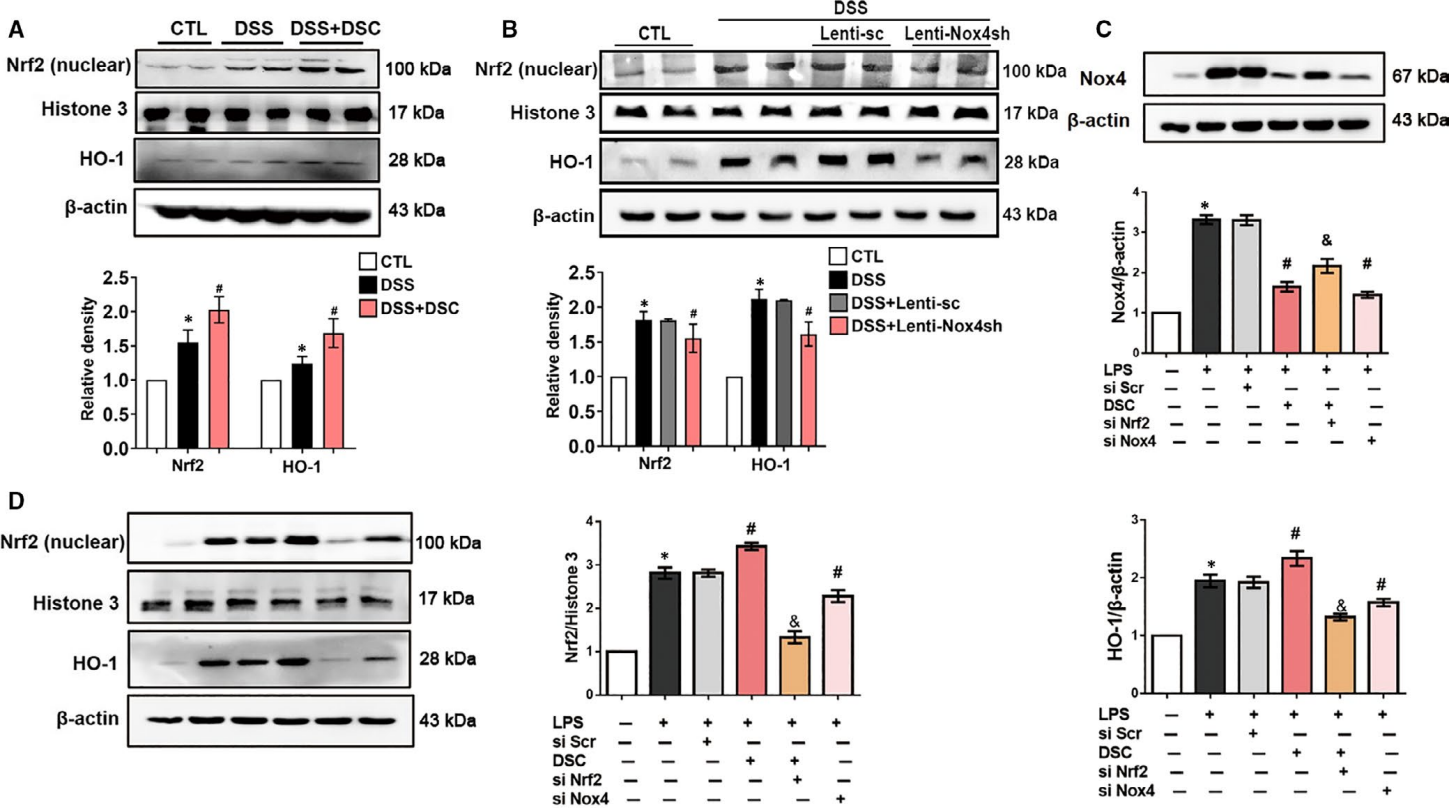
DSC restores redox balance in colonic tissues. A, Colitis was induced as described in Materials and Methods and treated with or without DSC (50 mg/kg). Representative bands and densitometry analysis of Nrf2 nuclear translocation and HO‐1 expression in colonic tissues. Histone H3 and β‐actin were used as loading control, respectively. B, Colitis was induced as described in Materials and Methods and treated with lentiviral Nox4 shRNA or lentiviral scrambled shRNA. Representative bands and densitometry analysis of Nrf2 nuclear translocation and HO‐1 expression in colonic tissues. Histone H3 and β‐actin were used as loading control, respectively. BMDM was stimulated with or without DSC (50 μmol/L) as described in Materials and Methods. C, Representative bands and densitometry analysis of Nox4. β‐actin was used as loading control. D, Representative bands and densitometry analysis of Nrf2 nuclear translocation and cytoplasmic HO‐1 expression in BMDM. β‐actin or histone H3 was used as loading control. Data shown are means ± SEM of n = 8 in each group. *P < 0.05 vs Control cell or mice (CTL), *^#^P* < 0.05 vs DSS‐treated mice or LPS‐stimulated cells

Our previous study reported that induction of Nrf2/HO‐1 might be, at least in part, responsible for the cytoprotective property of DSC.^26^ In the present study, the nuclear accumulation of Nrf2 and expression of HO‐1 was slightly increased in the colonic tissues of DSS‐induced mice. Correspondingly, DSC markedly induced Nrf2 nuclear accumulation and HO‐1 expression in colon tissues of DSS‐induced mice (Figure 7A). DSS‐mediated Nrf2 nuclear accumulation and HO‐1 expression was abolished by genetic Nox4 knockdown (Figure 7B). Consistent with in vivo results, both DSC treatment and Nox4 siRNA transfection markedly reduced LPS‐mediated Nox4 expression in LPS‐stimulated BMDM. However, the effects of DSC on Nox4 expression were reversed by Nrf2 silencing (Figure 7C). Conversely, Nox4 knockdown by siRNA markedly increased Nrf2 nuclear accumulation and up‐regulated HO‐1 expression in LPS‐stimulated BMDM (Figure 7D). Together, our data indicate that DSC restore redox homeostasis by down‐regulating Nox4 expression, contributing to suppressed inflammatory response.

## 4 DISCUSSION

Danshensu, a hydrophilic bioactive constitute of Danshen, attracts considerable interest due to its salubrious biological activities. We have earlier reported prominent anti‐oxidative and anti‐inflammatory activities with a novel danshensu derivative DSC in vitro and in vivo.^26^, ^29^, ^30^ In the present study, we clearly demonstrated that DSC suppressed DSS‐induced experimental colitis in vivo. Mechanistically, the anti‐inflammatory effects of DSC on inhibition of NF‐κB‐NLRP3 inflammasome activation were mediated via modulating Nox4‐mediated ROS signalling pathway. Our study suggests DSC mitigating Nox4 signalling as a novel therapeutic strategy for IBD.

Although the precise aetiology of IBD remains much debatable, there is now substantial evidence that excessive ROS is one of the most important aetiological factors involved in the pathophysiology of IBD.^40^ ROS plays pivotal roles in various biological activities, including inflammatory cytokine production, leucocyte infiltration and intestinal barrier dysfunction.^41^, ^42^ Ours as well as other previous studies have demonstrated that Nox4 plays a critical role in inflammatory diseases.^16^, ^21^, ^22^, ^23^, ^24^ Nox4 is mainly expressed in subcellular compartments like mitochondria, endoplasmic reticulum and plasma membrane. Nox4 constitutively generates ROS, and consequently, its activity reflects its level of expression.^21^ In the present study, we found that colonic Nox4 expression and ROS and H_2_O_2_ generation were significantly increased in DSS‐induced colitis mice, which was accompanied by increased inflammatory mediators and intestinal barrier dysfunction. Meanwhile, Nox4 expression and mitochondrial ROS and intracellular H_2_O_2_ production were significantly increased in nigericin‐activated LPS‐primed BMDM. Intriguingly, DSC treatment significantly mitigated DSS‐mediated Nox4 expression and colonic ROS and H_2_O_2_ generation and enhanced intestinal barrier integrity in vivo. Accordantly with the results obtained in vivo, we found that DSC inhibited Nox4 expression and subsequent mitochondrial ROS and intracellular H_2_O_2_ generation in vitro. Similarly, Nox4 knockdown also decreased LPS‐induced mitochondrial ROS and intracellular H_2_O_2_ production in BMDM. Meanwhile, our data demonstrated that genetic Nox4 knockdown also significantly attenuated colonic injury in DSS‐induced colitis. Collectively, our findings indicated a positive correlation of Nox4 levels with colitis and that modulation of Nox4 expression by DSC represented one possible mechanism of its beneficial effects on colitis.

Overproduction of ROS may stimulate NF‐κB activation, thereby leading to activation of NLRP3 inflammasome and release of IL‐1β and other inflammatory mediators including iNOS and COX‐2, which are associated with IBD.^14^, ^43^ Aberrant over‐activation of NF‐κB plays a critical role in the pathological process of IBD.^44^ In addition, Nox4‐derived ROS generation is one of common mechanisms of NLRP3 inflammasome activation, which may additionally contribute to accelerated inflammatory responses.^20^, ^23^ Phosphorylation of NF‐κB p65 (serine 536) controls the kinetics of NF‐κB nuclear translocation.^39^, ^45^ Meanwhile, phosphorylation of p65 at serine 536, but not other sites, appears to play a pivotal role in p65 DNA binding activity and transcriptional activity.^46^ In support of this, our results demonstrated that both DSC treatment and genetic Nox4 knockdown interfered with DSS‐induced Nox4 expression and NF‐κB activation in experimental colitis in mice. Additionally, we also observed that Nox4 silencing or DSC treatment significantly attenuated LPS‐mediated ROS generation and NF‐κB activation in inflammatory macrophages. NF‐κB is one key mechanism to mediate NLRP3 inflammasome activation, which may additionally contributes to accelerated inflammatory responses.^47^ Emerging evidence suggests the pivotal role of NLRP3 inflammasome in the development and pathogenesis of IBD and NLRP3 ^–/–^ mice were protected in experimental colitis.^48^ Consistent with these findings, we showed that DSC dramatically prevented NF‐κB activation and subsequently suppressed NLRP3 inflammasome activation (NLRP3, ASC, cleaved caspase‐1, cleaved IL‐1β and IL‐18) in vivo and in vitro. Taken together, these data supported that DSC alleviated experimental colitis at least in part through inhibiting Nox4‐mediated NF‐κB and NLRP3 inflammasome activation.

Loss of redox homeostasis plays a pivotal role in the development of IBD.^49^ The transcription factor Nrf2 is a master regulator of redox homeostasis.^50^ Nrf2 regulates the expression of a battery of cytoprotective genes, including HO‐1.^51^ We found that DSC significantly induced nuclear translocation of Nrf2 and increased HO‐1 expression in colonic tissues, associated with reduced ROS generation and NF‐κB activation. Our findings were consistent with previous reports that showed the protective effects of Nrf2 on the colitis are mediated by mechanisms involving ROS scavenging and/or inhibition of the NF‐κB pathway.^52^, ^53^ Aberrant Nox4 up‐regulation and the impairment to induce the Nrf2‐mediated antioxidant response indicated the involvement of oxidative stress.^54^ On the other hand, inducing Nrf2 activation is shown to inhibit Nox4 expression.^54^ Consistent with the previous report, we found that DSC activated Nrf2 with the suppression of Nox4 expression in vivo and in vitro. In LPS‐stimulated BMDM, the effects were abolished by Nrf2 siRNA. In addition, increasing evidence has suggested that ROS produced by NADPH oxidases can activate Nrf2 and induce HO‐1.^55^, ^56^ In LPS‐mediated inflamed BMDM, we showed that an aberrant up‐regulation of Nox4 was coupled with a Nrf2 induction, resulting in a sustained redox imbalance, which promoted inflammatory response. Interestingly, Nox4 siRNA knockdown markedly decreased ROS generation and subsequently down‐regulated Nrf2 nuclear translocation. The present data are consistent with the earlier report that Nox4 can activate the Nrf2‐regulated pathway through oxidative post‐translational modification.^57^ Taken together, Nrf2 and Nox4 reciprocally regulate the inflammatory response in DSS‐mediated colitis and LPS‐stimulated BMDM, and therefore, it is logical to believe that DSC restores redox homeostasis by regulation of the balance between Nrf2 and Nox4.

## 5 CONCLUSIONS

In summary, our study demonstrated that DSC alleviated DSS‐induced colitis by inhibiting Nox4‐mediated NF‐κB and NLRP3 inflammasome activation. Although the protective mechanism by which DSC modulates various signalling pathways remains to be fully understood, our findings suggest that DSC has promising therapeutic potential for IBD.

## CONFLICT OF INTEREST

The authors have declared no conflict of interest.

## AUTHOR CONTRIBUTION

**Li‐Long Pan:** Conceptualization (lead); Data curation (lead); Funding acquisition (equal); Investigation (lead); Writing‐original draft (lead); Writing‐review & editing (lead). **Zhengnan Ren:** Data curation (equal); Methodology (lead); Project administration (lead); Software (lead); Writing‐review & editing (supporting). **Yanyan Liu:** Investigation (equal); Methodology (equal); Project administration (lead); Software (equal). **Yalei Zhao:** Data curation (equal); Methodology (equal); Project administration (equal); Resources (lead). **Hongli Li:** Methodology (equal); Project administration (equal); Software (equal); Writing‐review & editing (supporting). **Xiaohua Pan:** Data curation (supporting); Investigation (supporting); Project administration (supporting); Writing‐review & editing (supporting). **Xin Fang:** Methodology (supporting); Project administration (supporting). **Wenjie Liang:** Methodology (supporting); Project administration (supporting). **Jun Yang:** Data curation (equal); Formal analysis (supporting); Methodology (supporting); Supervision (equal); Writing‐original draft (supporting); Writing‐review & editing (supporting). **Yang Wang:** Conceptualization (equal); Data curation (equal); Funding acquisition (equal); Writing‐review & editing (equal). **Jia Sun:** Conceptualization (supporting); Data curation (supporting); Funding acquisition (lead); Writing‐original draft (lead); Writing‐review & editing (lead).

## Supporting information

Supplementary Material

## Data Availability

The authors declare that the main data supporting the findings of this study are available within the article and its Supplementary Information. Extra data are available from the corresponding author upon request.
