# Supplementary material for: A novel danshensu derivative ameliorates experimental colitis by modulating NADPH oxidase 4‐dependent NLRP3 inflammasome activation
Source: J Cell Mol Med. 2020 Sep 17;24(22):12955–69. doi: 10.1111/jcmm.15890 (PMC7701520; doi:10.1111/jcmm.15890)

**Supplementary Information**

**Suppl. Tab. 1.** Primers used in real-time qPCR analysis.

| **Gene** | **Forward** | **Reverse** |
| --- | --- | --- |
| iNOS | 5’-GTTCTCAGCCCAACAATACAAGA-3’ | 5’-GTGGACGGGTCGATGTCAC-3’ |
| COX2 | 5’- CCCGGACTGGATTCTATGGTG-3’ | 5’-TTCGCAGGAAGGGGATGTTG-3’ |
| IL-1β | 5’-ATCTTTTGGGGTCCGTCAACT-3’ | 5’-GCAACTGTTCCTGAACTCAACT-3’ |
| IL-6 | 5’-TAGTCCTTCCTACCCCAATTTCC-3’ | 5’-TTGGTCCTTAGCCACTCCTTC-3’ |
| IL-18 | 5’-GGTCTCAACCCCCAGCTAGT-3’ | 5’-GCCGATGATCTCTCTCAAGTGAT-3’ |
| Nox1 | 5’-GTGATTACCAAGGTTGTCATGC-3’ | 5’-AAGCCTCGCTTCCTCATCTG -3’ |
| Nox2 | 5’- GACTGCGGAGAGTTTGGAAG-3’ | 5’-GGTGATGACCACCTTTTGCT-3' |
| Nox4 | 5’-GAAGGGGTTAAACACCTCTGC-3’ | 5’-ATGCTCTGCTTAAACACAATCCT-3’ |
| Ly6G | 5’-GACTTCCTGCAACACAACTACC-3’ | 5’-ACAGCATTACCAGTGATCTCAGT-3’ |
| F4/80 | 5’-TGACTCACCTTGTGGTCCTAA-3’ | 5’-CTTCCCAGAATCCAGTCTTTCC-3’ |
| ZO-2 | 5’-ACGACCGAGGTTTTGAAGTGA-3’ | 5’-CTGCGCCCATAGTCTTCCT-3’ |
| Caludin-1 | 5’-TATCCCAAGCCAACACCTTC-3’ | 5’-AGCCTTCCCTCCTGTGCT-3’ |
| Muc-2 | 5’-AGGGCTCGGAACTCCAGAAA-3’ | 5’CCAGGGAATCGGTAGACATCG-3’ |
| β-Actin | 5’-CCCAGGCATTGCTGACAGG-3’ | 5’-TGGAAGGTGGACAGTGAGGC-3’ |

**Suppl. Fig. 1.** DSC attenuates DSS-induced experimental colitis. Colitis was induced as described in Materials and methods and treated with indicated doses of DSC (12.5-100 mg·kg^-1^). (A) Body weight change, (B) DAI score, (C) colon length were determined as described in Materials and methods. Data shown are means ± SEM of *n=5* in each group. **P* < 0.05 compared with control mice, ^#^P < 0.05 compared with DSS-treated alone mice.


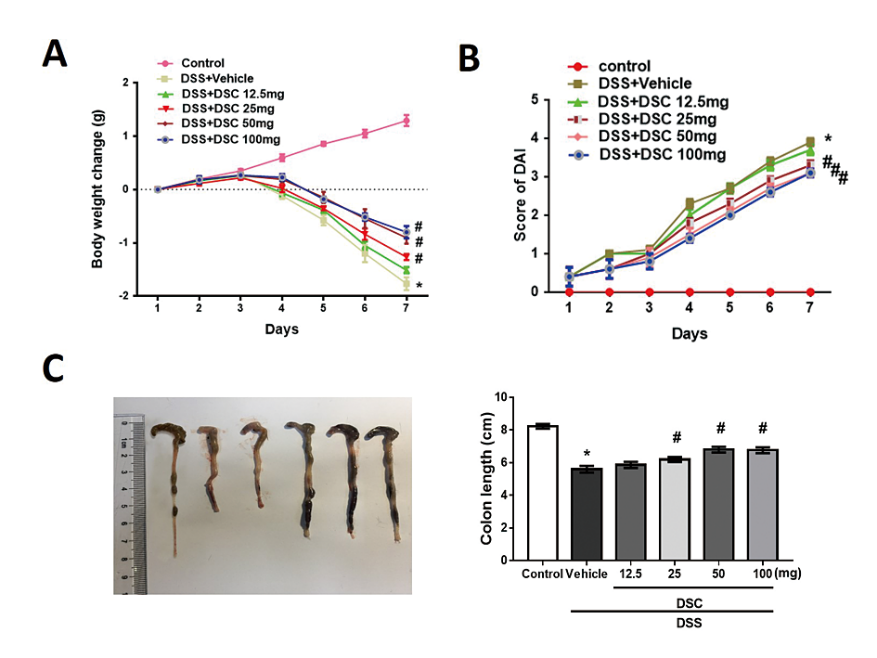


**Suppl. Fig. 2.** DSC reduces DSS-mediated infiltration of macrophage in colon. Colitis was induced as described in Materials and methods and treated with or without DSC (50 mg·kg^-1^). Representative images of macrophage infiltration by immunofluorescent staining in colonic tissues, scale bar =100 μm.


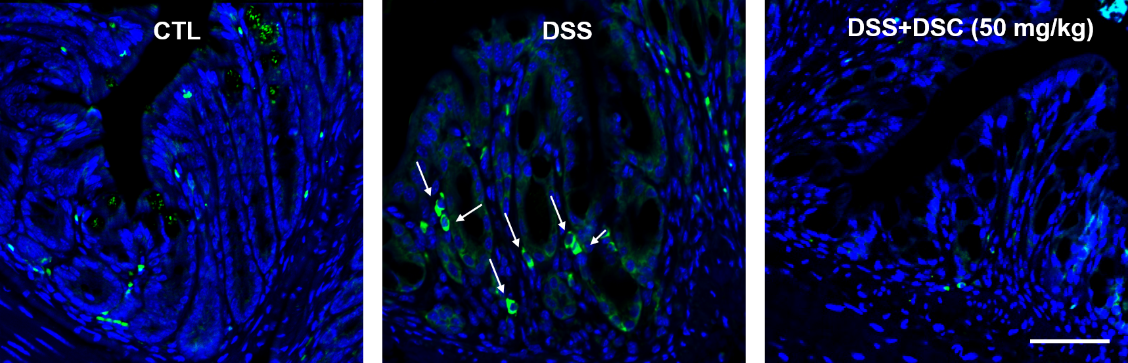


**Suppl. Fig. 3.** DSC reduces DSS-induced Nox4, but not Nox1 or Nox2 mRNA expression. Colitis was induced as described in Materials and methods and treated with or without DSC (50 mg·kg^-1^). The mRNA levels of Nox1, Nox2 and Nox4 were determined as described in Materials and methods. Data shown are means ± SEM of *n=8* in each group. **P* < 0.05.


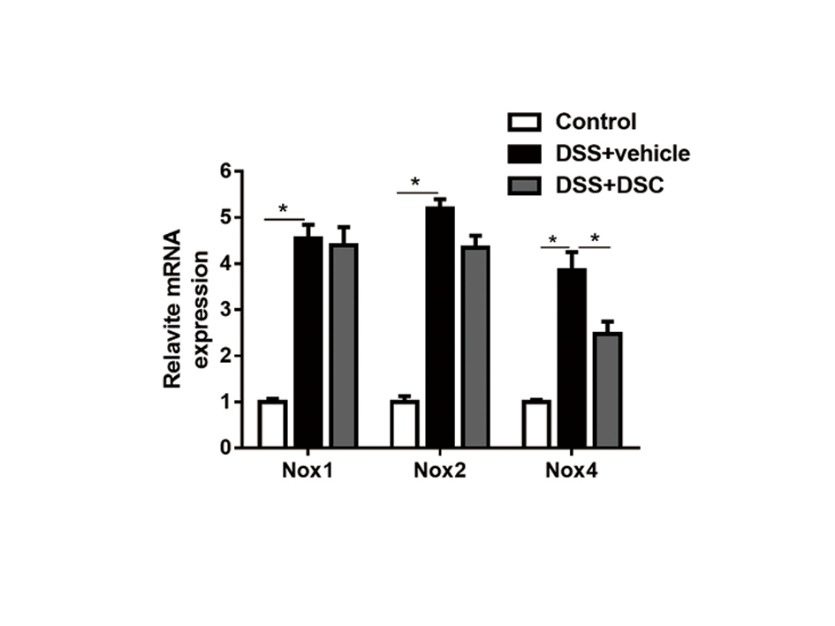


**Suppl. Fig. 4. DSC ameliorates colonic ROS production.** Colitis was induced as described in Materials and methods and the colonic tissues were collected as indicated periods. Representative images and quantitative analysis of ROS production by DHE staining in colonic tissues, scale bar =100 μm.


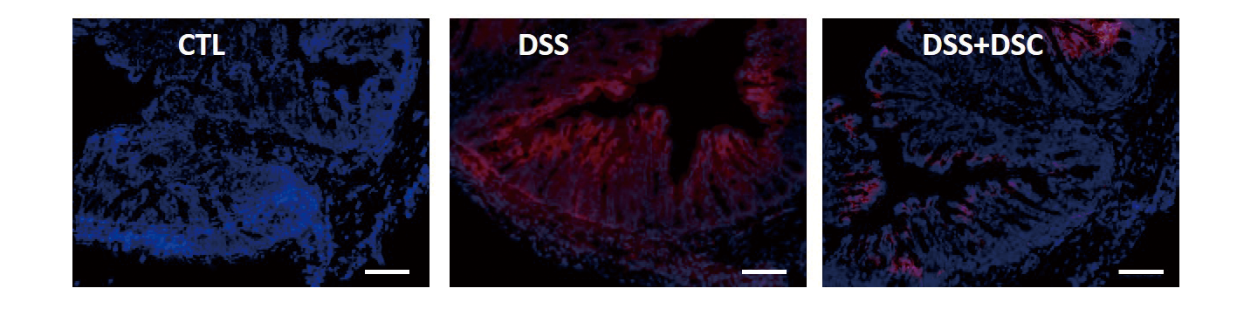


**Suppl. Fig. 5.** Lentiviral Nox4 shRNA knockdown protects against DSS-mediated colitis. Colitis was induced as described in Materials and methods and treated with or without lentiviral Nox4 shRNA. The body weight change (A), DAI score (B), histological score (C), MPO activities (D), H_2_O_2_ production (E), GSH/GSSG ratio (F) and inflammatory mediators (COX-2 and iNOS) were evaluated in colonic tissues after DSS administration. Data shown are means ± SEM of *n =8* in each group. **P* < 0.05.


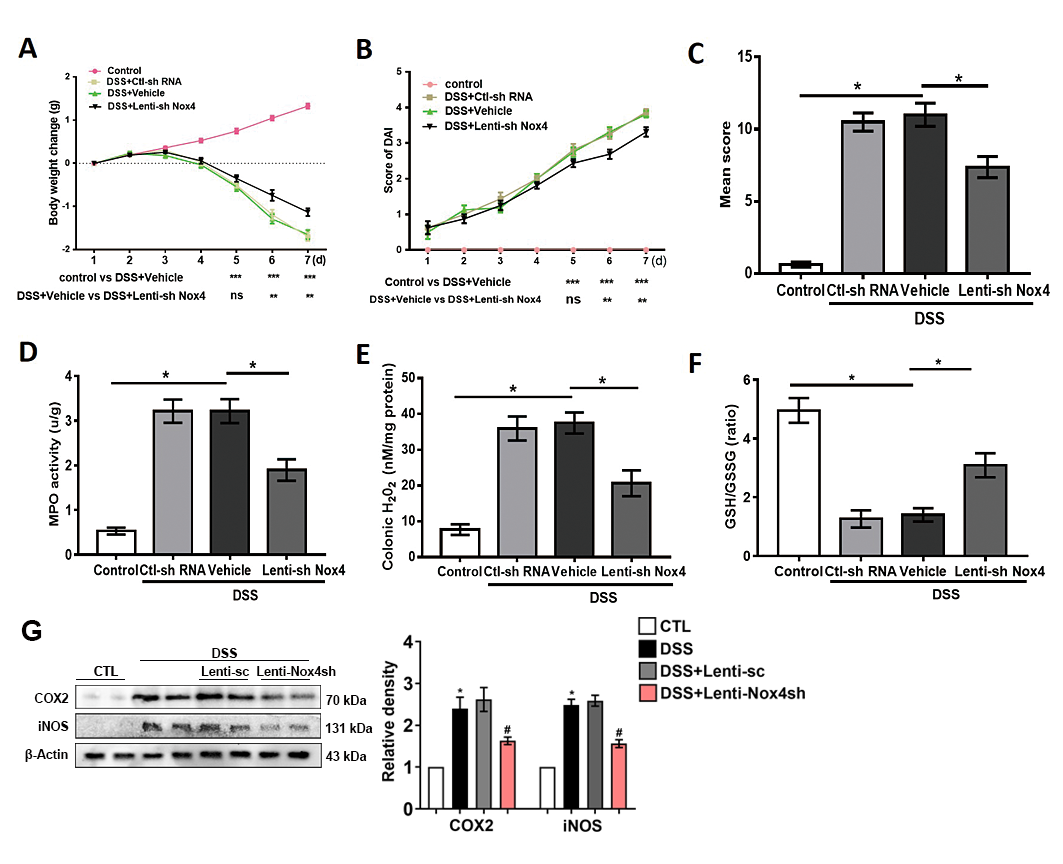


**Suppl. Fig. 6.** LPS time-dependently induces Nox4 expression in bone marrow-derived macrophages (BMDM). BMDM was stimulated with LPS (100 ng/ml) for the indicated time. The expression of Nox4 was analyzed by Western blot. Representative band and quantitative analysis of Nox4 in BMDM were shown and β-actin was used as loading control. Data shown are means ± SEM of *n=8* in each group. **P* < 0.05.


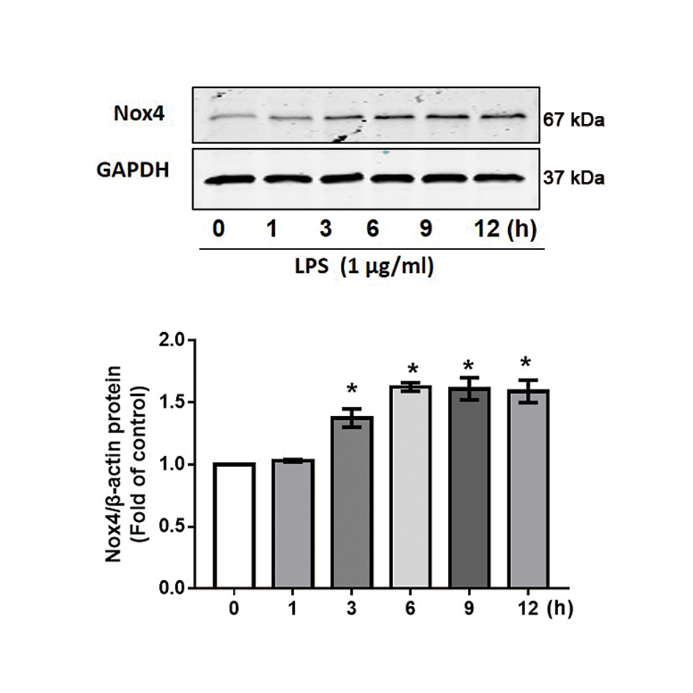

Supplement: Supplementary file 1 — Supplementary Material [file JCMM-24-12955-s001.docx]
